# Spatial health risk assessments of nickel in the groundwater sources of a mining-impacted area

**DOI:** 10.1038/s41598-024-61914-6

**Published:** 2024-05-14

**Authors:** Fatemeh Salehi, Milad Esmaeilbeigi, Ali Kazemi, Saeed Sharafi, Zahra Sahebi, Ali Ghanavati Asl

**Affiliations:** 1 Department of Environmental Science and Engineering, Faculty of Agriculture and Environment, Arak University, Arak, Iran; 2 Centre for Applied Water Science, Institute for Applied Ecology, University of Canberra, Canberra, ACT Australia; 3 Arvin Zist Pooya Lab, Tehran, 1563794747 Iran; 4 Researcher in Environmental Science and Engineering, Islamic Azad University, Ahvaz, Khuzestan Iran

**Keywords:** Groundwater, Vulnerable consumers, Hazard quotient, Risk assessment, South Khorasan, Non-carcinogenic risk, Environmental sciences, Health care

## Abstract

Mining activities have increased the potential risks of metal pollution to the groundwater resources in arid areas across the globe. Therefore, this study aimed to examine the health risk associated with nickel (Ni) in the groundwater sources of a mining-impacted area, South Khorasan, Eastern Iran. A total of 110 stations were included in the study, comprising 62 wells, 40 qanats, and 8 springs in summer, 2020. Initially, the collected samples were tested for temperature, pH, and electrical conductivity (EC). Subsequently, the samples were filtered and treated with nitric acid (HNO_3_) to measure the concentration of Ni using Inductively Coupled Plasma Mass Spectrometry (ICP-MS). Hazard quotient (HQ) and non-carcinogenic risk assessments were employed to evaluate the potential risks of Ni to the inhabitants. The findings revealed that the concentration of Ni ranged from 0.02 to 132.39 μg l^−1^, and only two stations exhibited Ni concentrations above the WHO standards (20 μg l^−1^). The results demonstrated that 98.21% of the sampled locations had HQ values below one, indicating negligible risk, while 1.78% of the stations exhibited HQ values of one or higher, representing a high non-carcinogenic risk for water consumers. Overall, the concentration of nickel in the groundwater of South Khorasan exceeded the World Health Organization (WHO) limit solely in the Halvan station, posing a non-carcinogenic risk for the residents in that area, and therefore, additional efforts should be made to provide healthier groundwater to consumers in this region.

## Introduction

A reliable and sufficient water supply is crucial for sustainable human development and overall well-being. However, uncertainties related to future climate change are raising concerns about the already limited freshwater resources available^1^. The global demand for water resources is increasing by 1% annually due to population growth, economic development, and changing consumption patterns^2^. Water scarcity presents a significant challenge for both developed and developing nations around the world and is further compounded by population growth and increasing global temperatures^3^. The lack of treated potable water will extend beyond surface water sources such as groundwater, rivers, streams, and wells in the future. Studies conducted by the FAO in 93 countries indicate water instability in these regions, where water use exceeds its replenishment.

Groundwater is of utmost importance due to its natural availability and potential to meet increasing demands as a substitute for surface water^4^. Globally, groundwater resources constitute a significant portion of the world's water supply^5^. Iran, with its unique geographical location, scattered topography, and air mass, is classified as one of the world's arid regions, relying on groundwater sources for over 90% of its freshwater supply^6^. The underground systems such as qanat, despite their old structure, are still being used to supply fresh water in such arid regions Therefore, the harvested water via underground systems such as qanat, spring, and well contain many toxic contaminants, including nickel that cause health-related problems in consumers. The presence of heavy metals in soil and water poses threats to aquatic ecosystems, agriculture, and public health. The accumulation of heavy metals in the food chain can significantly contribute to carcinogenesis, mutagenesis, and the birth of malformed babies^7^. Prolonged exposure to heavy metals, which can exist in toxic concentrations in water, has resulted in severe health damage to vital organs such as the brain, liver, bones, and kidneys, where they tend to accumulate. Additionally, when concentrations exceed the permissible levels set by the World Health Organization (WHO), heavy metal exposure disrupts metabolic processes in the body^8^.

Ni is a prevalent metal found in food and drinking water from both natural and human-related sources. Oral exposure to Ni can lead to various adverse effects^9^. Analyzing risk as an assessment can provide environmentalists with valuable insights into understanding the carcinogenic and non-carcinogenic effects of metals on human health. Research conducted by Kazemi et al. suggests that hygiene agencies should be encouraged to ensure clean water for consumers^10^. As a result, numerous researchers worldwide have been actively engaged in this field. For instance, a study focused on groundwater pollution and associated risks to human health was conducted near the ophiolitic complex. It evaluated heavy metals such as Fe and As, revealing significant pollution in the groundwater^11^. Likewise, another study conducted in eastern Iran analyzed the accumulation of heavy metals in soil and water. The results indicated that, except for arsenic, antimony, and iron, the levels of metals examined in soil samples were lower than the global concentration. Furthermore, the chemical analysis of water samples, compared to international standards, confirmed contamination by heavy metals, rendering the water sources undrinkable in that region^12^. In a hydro-geochemical investigation conducted in an area of Izmir known for its economic natural mineral reserves, the levels of Al, Ni, and Sb in groundwater exceeded the drinking water standards^13^.

South Khorasan boasts a wide range of mines, including stone, cement, coal, and metals, which significantly contribute to Iran's gross domestic product^14^. Groundwater, particularly qanat in South Khorasan, serves as a vital water reservoir for drinking purposes, and residents continue to rely on it. Because of this, it is crucial to evaluate the quality of groundwater resources for the well-being of the residents. Considering the significance of assessing heavy metal concentrations in groundwater and ensuring the health of the residents in this area, the present study aims to investigate if TNi could cause health-related issues in levels in underground waters of South Khorasan and evaluate the health risks for adults using non-carcinogenic methods. Therefore, we aimed to answer the questions (i) how the spatial distribution health risks associated with Ni differ in different locations across this mining-impacted area, and (ii) how the contamination of groundwater with Ni in this area compares to the international standard limits.

## Materials and methods

### Study area

**Figure 1:**
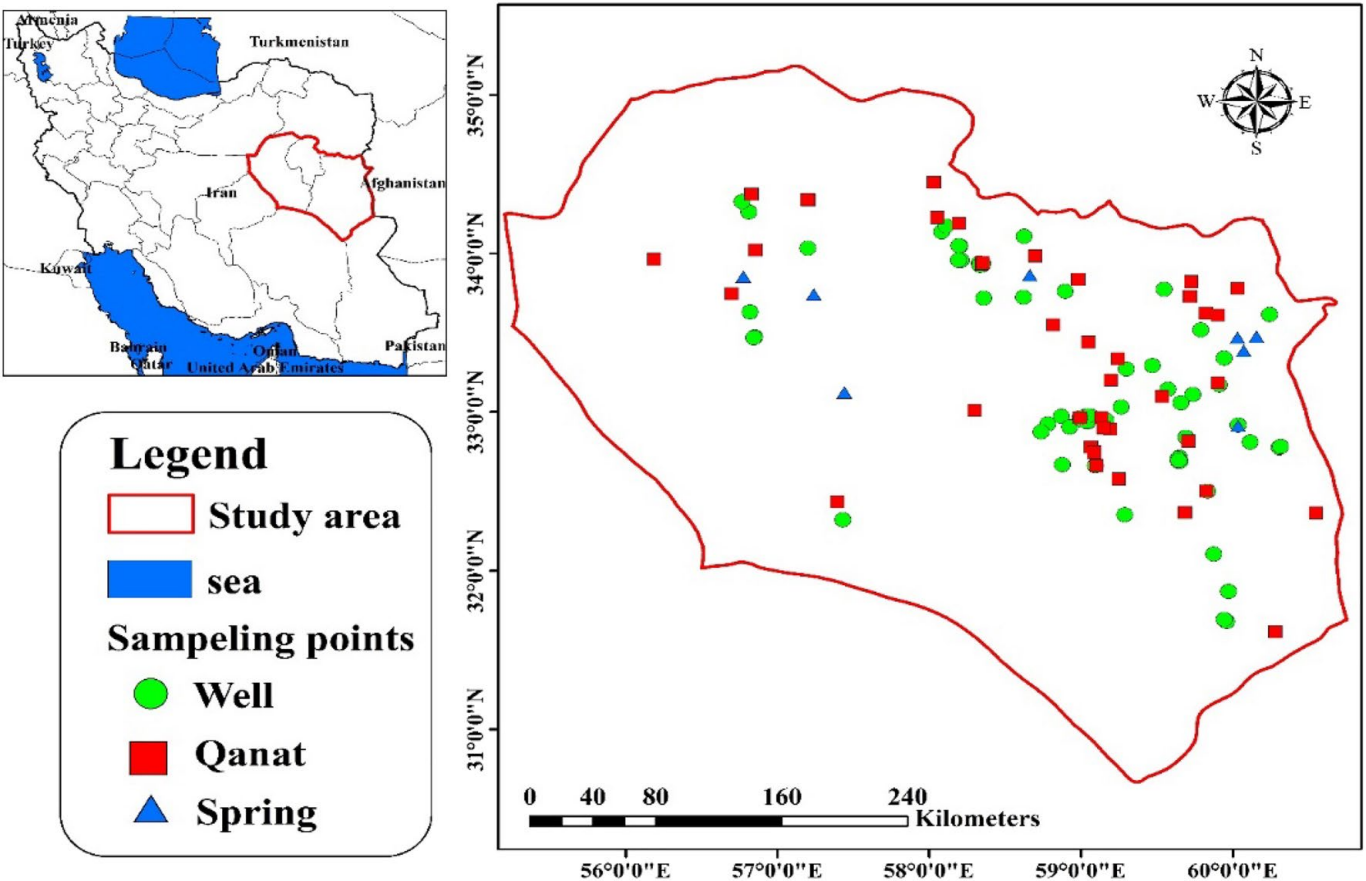
Location of sampled groundwater in South Khorasan, east Iran. The map was created by created by ArcGIS (version 10.3; ESRI Inc., Redlands CA).

Eastern Iran possesses a significant number of underground sources, serving as a vital source of drinking and agricultural water due to the region's arid conditions and lack of surface water. The South Khorasan province centered around the city of Brigand, spans an area of 62,666 square meters, ranging from 31 degrees 32 min to 31 degrees 51 min north latitude from the equator and 52 degrees 52 min to 61 degrees 52 min east longitude from the Greenwich meridian. South Khorasan is located at the latitude and longitude coordinates of 32.5176° N to 59.1042° E, covering approximately 151,913 km^−2^. Within this area, approximately 6, 250 qanats have been registered. A land use map was created using ArcGIS software (version 10.3; ESRI Inc., Redlands CA) to depict the distribution of different areas and the groundwater explored in this study (Fig. 1). The majority of the surveyed region consisted of agricultural and industrial lands. Within this figure, various symbols indicate the different types of stations, including wells, qanats, and springs, each represented separately.

### Sample collection and storage

A number of 110 groundwater stations (62 wells, 40 qanats, and 8 springs) located in cities, plains, and rural areas were sampled in triplicate (each sampling location with three samples) to assess the Ni content within the study area in the summer of 2020. Qanat is an ancient water supply system in many arid and semi-arid areas in Asia, especially in the Middle East. This historical underground system has been playing a crucial role in supplying fresh water in Eastern Iran, where many cities are suffering from enough surface water. The selection of these stations was based on factors such as population density, environmental pollutants, and industrial activities. Geographical features, groundwater flow, and population density were taken into consideration during the sampling process. To measure temperature, pH, and electrical conductivity (EC) at the sampling locations, a portable multi-parameter instrument (SensoDirect 150, Lovibond, Germany) was employed. Subsequently, the samples were collected in polypropylene containers, and a few drops of nitric acid (about 500 µL HNO_3_ 65%) were added into each container in situ to stabilize the samples. Samples were appropriately labeled and placed in an icebox for transportation to the lab within 24 h for analysis. Upon arrival at the laboratory, the water samples were transferred to a refrigerator and stored at 4 °C until the commencement of the laboratory analysis. In the lab, samples were analyzed for Ni concentrations using an Inductively Coupled Plasma Mass Spectrometry (ICP-MS) (HP-4500 USA, equipped with autosampler Asx-520) machine in triplicate. For accuracy, limits of detection (LOD) and relative standard deviation (RSD) were examined (below 5% for all samples) using standard methods, and LOD demonstrated a value of 1ng L^−1^ for Ni.

### Health risk assessment

Groundwater contamination can have severe implications for human health and sustainable social development. The primary goal of risk assessment is to safeguard the health of individuals who are exposed to water, either directly (such as through drinking water) or indirectly (such as consuming food irrigated with water), as well as protect aquatic ecosystems (Agbasi et al. 2023). Humans can be exposed to heavy metals through various means, including inhalation via the mouth and nose, contact with the skin and ingestion. These routes of exposure are thoroughly examined in toxicological studies. In particular, ingestion plays a significant role in human exposure to toxins, as highlighted by the Environmental Protection Agency (EPA) in 1989. To determine the non-carcinogenic average daily dose (ADDnc) for nickel intake, calculations were performed using Eq. 1, as per Ghahramani et al.^15^ specified by the EPA in 1989. Constant numbers and input values required for the calculations are given in Table 1.

1 $$ADD_{nc} = \frac{{C \times IR \times EF \times ED_{nc} }}{{BW \times AT_{nc} }}$$

**Table 1:** Constant numbers and input values used for ADD_nc_ and HQ calculations.

Factor (for Ni)	Value	Definition	References
RfD (oral) (μg kg^−1^ day^−1^)	20	Reference dose	Vahidifar et al.^5^, Mohammadi et al.^3^
C (μg l^−1^)	Based on water samples	Ni concentration	–
IR (l day^−1^)	2 for adults	Ingestion rate	Kazemi et al.^10^
EF (day year^−1^)	365	Exposure frequency	
ED (year)	70 for adults	Exposure duration	
BW (kg)	72 for adults	Body weight	
BW (kg)	14.5 for children	Body weight	Vahidifar et al.^5^
AT (day)	Lifetime (70 years) × 365 days per year = 25,550	Average time cancer	Mohammadi et al.^3^

### Non-carcinogenic risk assessment

HQ is used to estimate the health risks of water consumption for humans as per Reddy and Sunith^16^ and EPA (1989). In this equation, ADD_nc_ is the average daily concentration of metals with a non-carcinogenic capacity based on (μg kg^−1^ day^−1^), and RFD is considered as a reference dose (μg kg^−1^ day^−1^) (Eq. 2).

2 $$HQ = \frac{{ADD_{nc} }}{RFD}$$

### Hazard quotient

For hazard quotient, HQ ≥ 1 showed the presence of non-carcinogenic risk, and HQ < 1 represented negligible hazard. For all 110 stations, the value of HQ was evaluated.

### Statistical analysis

In this study, descriptive statistics were calculated to determine the average, minimum, maximum, standard deviation, and range of Ni concentrations in the studied stations. To examine potential correlations between Ni concentration and HQ (Hazard Quotient) values in three types of groundwater (well, spring, and qanat), Pearson's correlation analysis was conducted using SPSS version 16 and significance of differences were showed at 0.01 level. Non-carcinogenic risk assessments were performed using standard models defined in Microsoft Excel software (Version 2016, Windows 10). Geographical images and maps were generated using ArcGIS (version 10.3; ESRI Inc., Redlands CA).

### Ethical responsibilities of authors

All authors have read, understood, and complied with the ethical responsibilities of authors as found in the instructions for authors in this journal.

## Results and discussion

### Physicochemical characteristics of groundwater

The physicochemical parameters of groundwater samples were analyzed at 110 different sites, comprising 62 wells, 40 qanats, and 8 springs (Table 2). While pH does not directly influence consumer health, it serves as a crucial indicator of water quality. The pH levels of the water samples were measured on-site and fell within the range of 6.62 to 8.63, with an average of 7.9. Except for the Giv station, all stations exhibited a neutral pH level, adhering to the permissible limits set by the WHO and the United States Environment Protection Agency (USEPA) (6.5–8.5)^17^. The DigRostam well station recorded the lowest pH at 6.62, while the Giv qanat station exhibited the highest pH at 8.63.

**Table 2:** Physicochemical parameters of groundwater in sampled locations.

Parameters	Type	Mean	Maximum	Minimum	Range
pH	Well	7.85	8.40	6.62	6.62–8.40
	Qanat	8.02	8.63	7.45	7.45–8.63
	Spring	7.88	8.46	7.55	7.55–8.46
EC	Well	2.00	5.73	0.79	0.79–5.73
	Qanat	1.72	6.73	0.37	0.37–6.73
	Spring	0.84	1.80	0.33	0.33–1.80

The electrical conductivity (EC) measurements across all areas ranged from 0.33 to 6.37 (mμ cm^−2^). The Gazdez region recorded the highest EC value of 6.73. The physicochemical parameters of groundwater depend on both natural (e.g. geology and minerals) and human-based factors (e.g. agriculture and mining). Chronic water shortages, with an annual average rainfall of less than 100 mm in this region have led to increasing changes in the hadrochemical parameters in the groundwater^18,19^. However, most of the studied sites showed the values for the water quality within the permissible range of WHO and USEPA.

### Ni concentrations and standard limits

The concentrations of Ni in 110 stations were assessed and compared to the standard values set by the Canadian Standard and the Environmental Protection Organization of Iran, which are 25 μg l^−1^ and 20 μg l^−1^, respectively. In the qanat water samples, the concentration of Ni varied between 0.02 and 132.39 μg l^−1^, with an average concentration of 5.09 μg l^−1^ across all studied qanats (Table 3). Among all the stations, Halvan qanat had the highest concentration at 132.39 μg l^−1^, followed by Grymanj at 24.37 μg l^−1^. Mining activities have had a significant impact on the surface and groundwater conditions over the past century^20^. Water pollution caused by heavy metals in abandoned mines is a critical environmental issue, particularly in arid and semi-arid regions where water resources are limited^21^. Heavy metal pollution in water is considered as one of the most serious environmental problems globally^7^. In studied area, it is highly possible that metal pollution causes by the permeation from the mining sites to the rock soil and then groundwater. Ni in low concentrations has no adverse effects on the human health, but exceeding concentration of Ni could cause health risks in target consumers. USEPA suggested an acceptable concentration for Ni at 100 μg l^−1^, while we found the highest Ni concentrations at 132.39 μg l^−1^ in Halvan station, where mining activities have increased over the last three decades. Kazemi et al. stated that there is a relationship between the density of active mines and groundwater pollution, meaning that the concentrations of Ni in the groundwater of heavily mining sites are higher than the areas with fewer mines^22^. In our study area, the mean spatial distance between sampling sites which high Ni concentrations and abandoned mines were at 7.8 ± 1.79 km, and locations with the highest Ni concentrations showed the lowest spatial distance with the mines. Having a long horizontal water channel underground, water in qanats and springs experiences many geochemical changes, including dissolution of metals and salts. Ni is more mobile in the acidic waters relative to the alkaline conditions. Therefore, one reason for the higher Ni concentrations in some of our sites might be due to the presence of acid-intensifying factors (e.g. soil and rock).

**Table 3:** Mean Ni concentrations in the water (μg L^−1^) sampled from a total of all stations.

Stations	Min	Max	Range	Average	SD
Well	0.02	2.97	0.02–2.97	0.64	0.682746
Qanat	0.02	132.39	0.02–132.39	5.09	21.35404
spring	0.06	3.33	0.06–3.33	1.12	1.113492

### Non-carcinogenic risk assessment of Ni

**Figure 2:**
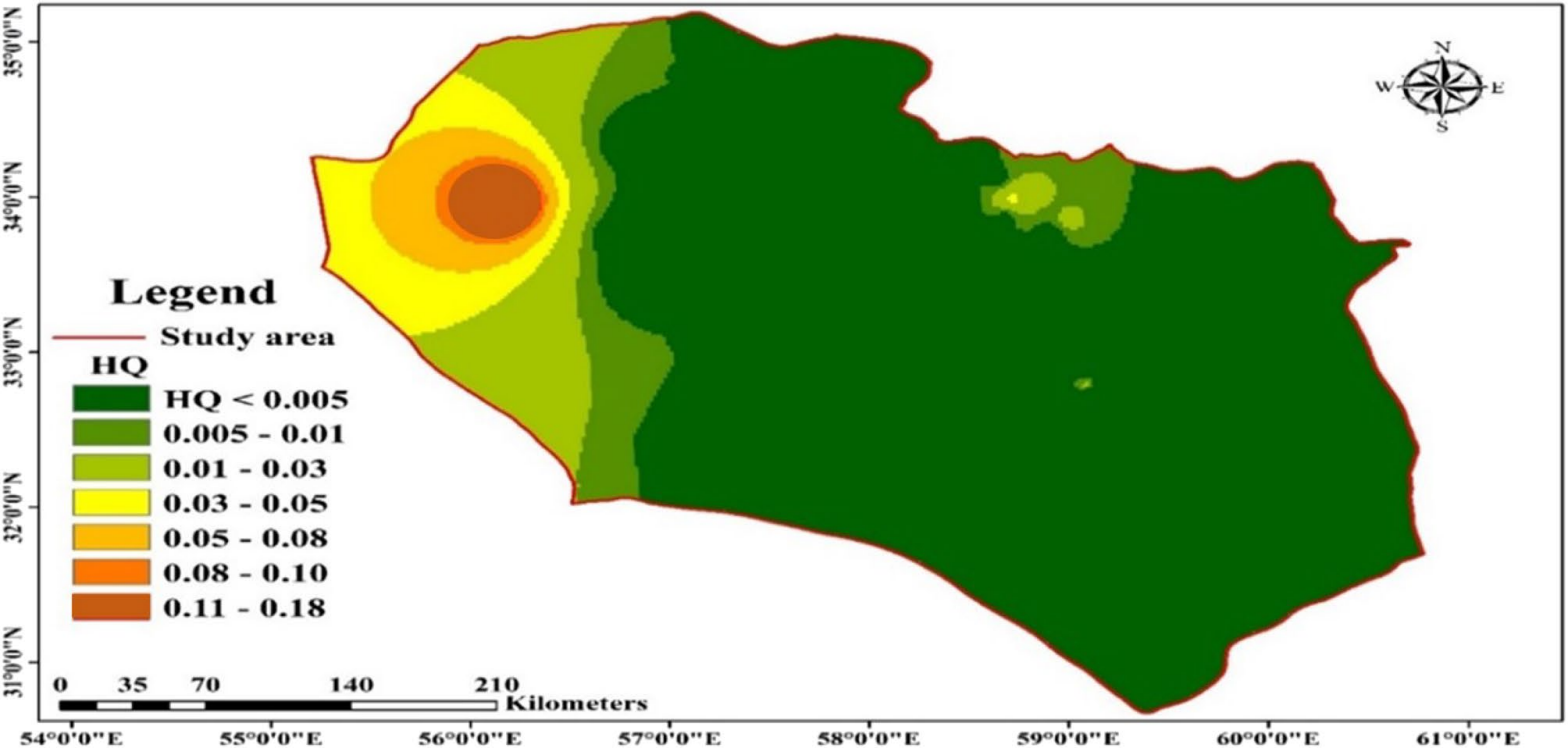
Zoning map of non-carcinogenic risk assessment of Ni in the study area created by ArcGIS (version 10.3; ESRI Inc., Redlands CA).

**Figure 3:**
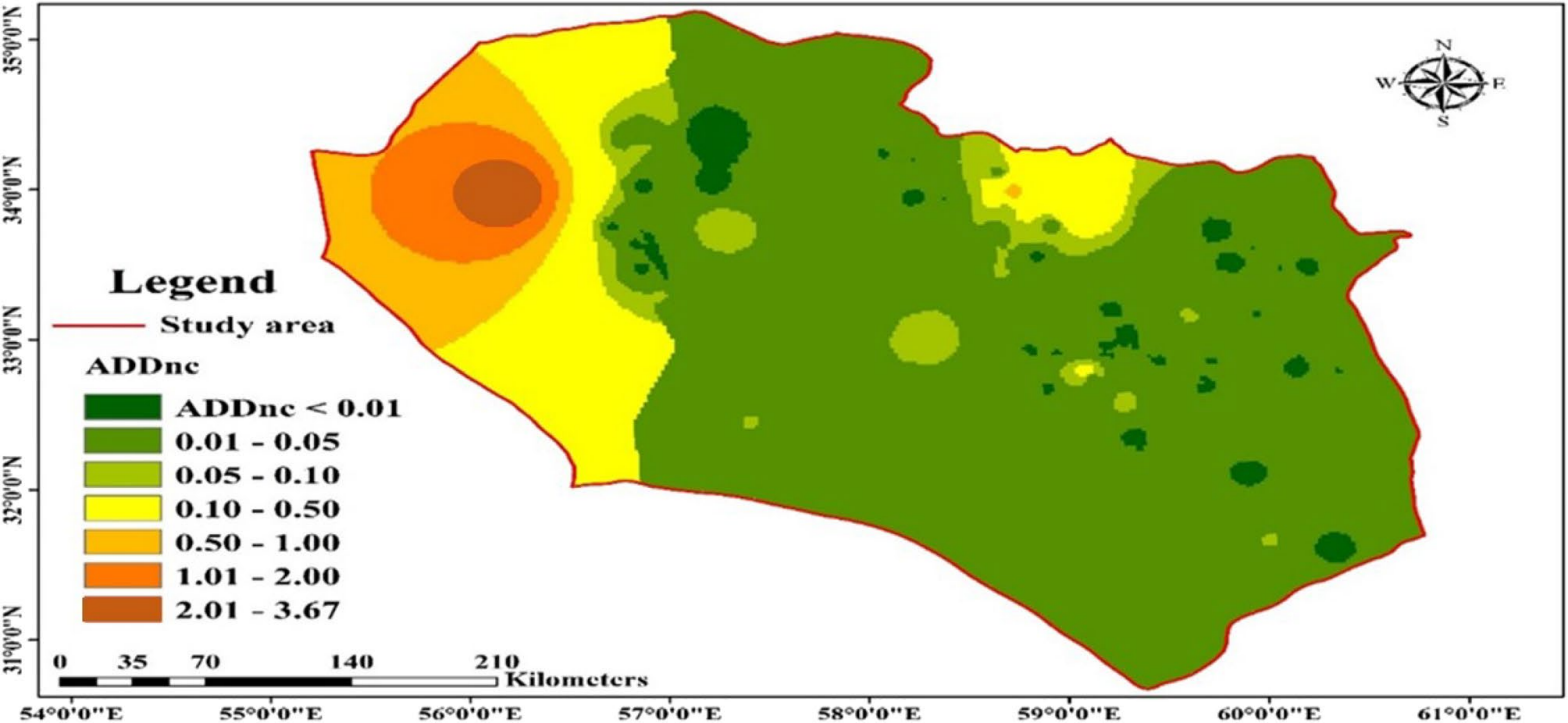
Zoning map of non-carcinogenic average daily dose (ADDnc) of Ni concentration in the study area created by ArcGIS (version 10.3; ESRI Inc., Redlands CA).

The findings of the non-carcinogenic risk assessment for Ni indicate that only the Halvan station, with a hazard quotient (HQ) value of ≥ 1, poses a potential non-carcinogenic risk to the residents. Conversely, the remaining sites, with HQ values < 1, present a negligible risk. The distribution of non-carcinogenic risk across the studied sites plotted using the zoning map created using ArcGIS (v) (Fig. 2). Additionally, non-carcinogenic average daily dose (ADD_nc_) for each of the sampled groundwater sites was mapped using ArcGIS (Fig. 3). Assessing the risk of groundwater contamination is an essential component of evaluating groundwater quality to the consumers. Albint introduced the concept of groundwater vulnerability and focused on the sensitivity of groundwater to changes resulting from human activities and natural incidents^23^, emphasizing the adaptability of the groundwater environment^24^. In line with our results, Ganiyu et al. showed the concentrations of heavy metals (e.g., Zn, Fe, and Pb) were at the highest levels in the water samples collected from the shallow wells with the lowest risk to adults in Southeast Nigeria^25^. Elevated levels of toxic metals in water are usually associated with industrial, mining, and agricultural operations. Ni, the 23rd most abundant element in the earth's crust, is among these toxic metals^26^. Acute exposure to Ni can alter the gene expression of normal cells, increasing the risk of Ni-induced cancers. Ni also contributes to the development of malignant properties in cells^27^. It is used in steel and other alloys, water treatment, batteries, and acts as a catalyst)^26^, therefore, Ni is ubiquitous in the environment and threats people’s health.

Our findings indicated that the concentration of Ni exceeded the standards in 5.5% of the sampled qanats, particularly in the Halvan and Grymanj stations. The HQ analysis revealed that 98.21% of the samples posed negligible risks (HQ < 1), while 1.78% of stations presented non-carcinogenic risks to consumers (HQ ≥ 1). The northwestern regions of South Khorasan exhibited the highest severity of non-carcinogenic risks associated with Ni contamination. Conversely, the western areas did not show any significant non-carcinogenic effects related to Ni. This difference can be attributed to the increasing mining and industrial activities in South Khorasan, leading to metal pollution in these areas. For instance, the Halvan region, located 80 km away from Tabas, is in proximity to various active mines in the Tabas region, which may explain the high Ni concentration in the Halvan station. Furthermore, the analysis of physicochemical parameters indicated that the areas with the highest pH generally exhibited the highest Ni concentrations. However, Pearson's statistical correlation calculation did not show a significant relationship between Ni and pH^22^. In a similar study conducted by Rezaei et al. in Sistan and Baluchestan, which almost the similar climate condition to that of our study area, the concentrations of heavy metals exceeded the acceptable limits stated by the international standards, with most of the sampling locations being affected by the agricultural activities and fertilizers^28^.

With the growing concern for drinking water quality, extensive studies worldwide have been conducted to address the decline in water quality. For example, in a study conducted by Rajaei et al. on the physicochemical analysis of irrigation and drinking water sources in the South Khorasan province^29^. The analysis of water samples from various stations in South Khorasan revealed that the electrical conductivity (EC) varied from 0.33 to 6.37 mS/cm, with an average of 1.78 mS/cm. The station with the lowest EC was Gazakht (agricultural land), while the highest EC was recorded at Gazdez station. pH analysis indicated that the Giv station had the highest pH, while the Gezdez station had the lowest pH. Only two of the qanats exceeded the standard limit of 20 μg/l for drinking water, as declared by the World Health Organization (WHO). The concentrations of these qanats were measured at 132.39 μg/l and 24.37 μg/l, respectively^30^.

Furthermore, the health risk assessment based on the hazard quotient (HQ) revealed that 98.21% of the studied qanats had HQ values below 1, indicating a non-carcinogenic risk to consumers. Conversely, 1.78% of the qanats exhibited HQ values equal to or exceeding 1, suggesting a potential non-carcinogenic risk. Pearson tests conducted on the data showed a strong and significant relationship (at the 0.01 level) between the nickel concentration in each area and the HQ value of the corresponding point across all the studied stations in South Khorasan, including wells, qanats, and springs (Table 4). In response to this crisis, the utilization of groundwater reservoirs has become a crucial strategy for accessing untapped water resources^31^. While the exploitation of mineral resources has been essential for human development, it has also led to detrimental environmental impacts, often wrongly attributed to natural causes, posing a threat to both aquatic and terrestrial ecosystems^32^. Mining operations, throughout the lifecycle of a mine and even after its closure, have a significant impact on water sources, including surface and groundwater. Processes such as extraction, flooding, dewatering, and the discharge of untreated water are all associated with water pollution^33^. Our findings are also in agreement with our previous study that we conducted on three different underground resources in South Khorasan as the health risk associated with metals increased with the density of mining activities and development^10^. Given the prevalence of mining activities in this area, there is a possibility of groundwater contamination by toxic and carcinogenic metals, resulting from soil and underground rock pollution. Despite the risks, people in South Khorasan still rely on groundwater for drinking, industrial use, agriculture, and livestock due to its quality and mineral content. However, the abundance of mining activities increases the potential for groundwater sources to become contaminated by toxic and carcinogenic metals seeping through the soil and rocks of the aquifer.

**Table 4:** Pearson correlation analysis of Ni concentration and the associated HQ in the sampling locations.

Test	Ni vs. HQ in well	Ni vs. HQ in qanat	Ni vs. HQ in spring
Pearson correlation	1.00**	1.00**	1.00**
Sig. (2-tailed)	0.000	0.000	0.000
N	62	40	8

**Correlation is significant at the 0.01 level (2-tailed).

## Conclusion

Eastern Iran is renowned for its abundant mineral resources and thriving industrial activities, particularly in South Khorasan. However, such developments have led to increasing the risk of environmental pollutants to the people’s life. In our study, the concentration of nickel in most of the investigated groundwater samples was within acceptable limits, posing no significant risks except for Halvan and Grymanj sites, where the levels of nickel contamination were potentially harmful and dangerous. Considering an ever-increasing industrial development in South Khorasan, this area has been facing many mining-based issues, including the risk of metal pollution in the water resources. Although, Ni contamination mostly could be due to the mining activities, other sources such as agriculture and natural incidents could also contribute to the Ni distribution. Children and adults are the most vulnerable consumers to the metal pollution and Ni concentrations more than the recommended values (e.g. USEPA, 100 µg L^−1^) cause non-carcinogenic risks in South Khorasan. In conclusion, groundwater resources in the study area are contaminated by Ni and in some sites, it could potentially cause health related issues to vulnerable consumers, therefore, the quality of water should be regularly monitored to avoid threatening the vulnerable resident’s health in this region.

## Data Availability

The authors confirm that the data supporting the findings of this study are available within the article.
